# Effect of Smoking on the Healing of Tibial Shaft Fractures in a Rural Indian Population

**DOI:** 10.7759/cureus.23018

**Published:** 2022-03-10

**Authors:** Nandini Sanjay, Arun H Shanthappa

**Affiliations:** 1 Department of Orthopaedics, Sri Devaraj Urs Academy of Higher Education and Research, Kolar, IND

**Keywords:** nonunion, infection, fracture healing, smoking, tibia

## Abstract

Background

Tibial shaft fractures account for 17% of all lower limb fractures. Nonunion and infection rates are estimated to be between 2% and 10%. Bone healing is a complex process that is influenced by biological, mechanical, and systemic factors. Adverse smoking effects on cardiovascular and respiratory systems have been well documented. An increasing interest in the effect of smoking on fracture healing following trauma has been noted in recent years. The biological consequence of smoking is relevant, especially in trauma surgery where no way of preventing presurgical smoking has been noted, hence increasing the patient’s risk of nonunion. Cigarette smoking has been shown to impair fracture union and wound healing and lead to an increased risk of fracture site infection. Smoking and high-energy trauma are considered important risk factors for the delayed union of tibial shaft fractures.

Objectives

This study aims to assess the adverse effects of smoking in patients with tibial shaft fractures following trauma and fracture fixation.

Materials and methods

A retrospective cohort study was done on 110 (55 smokers and 55 nonsmokers) patients treated with intramedullary nailing or plating for tibial shaft fractures between July 2017 and January 2021 in the hospital of the current study. Fracture healing was assessed at the end of months 1, 3, and 6 and year 1.

Results

The mean time of healing in smokers was >48 weeks, whereas the average time to union was 24 weeks in nonsmokers. The majority (54.6%) of smokers took >48 weeks to heal, whereas 81.8% of patients in the nonsmoking group took 24-28 weeks to heal.

Conclusion

Similar to the results obtained in previous studies, our study showed that smoking hinders fracture healing after surgical fixation, and smokers have a higher chance of developing surgical site infection and osteomyelitis. Smokers take a longer time for radiological union and also have a high chance of delayed union and nonunion when compared with nonsmokers, which was shown in our study and is consistent with the results obtained in previous studies. Postoperative smoking cessation is as important as preoperative smoking cessation, and patients should be strictly counseled regarding the same.

## Introduction

Tibial shaft fractures are the most common long bone fractures [1]. Although intramedullary nailing is the gold standard for the treatment of tibial shaft fractures, the minimally invasive percutaneous plate osteosynthesis (MIPO) technique has now become popular for treating tibial shaft fractures, especially distal third tibia fractures [2]. Bone healing following a fracture is a complex process that is influenced by several factors. Primary bone healing occurs without callus formation. Secondary bone healing occurs with callus formation.

Factors interfering in the aforementioned process are categorized into local and systemic factors. Local factors include excessive movement at the fracture site, infection, and decreased blood supply to the fracture site. Systemic factors include obesity, diabetes, malnutrition, and smoking [3]. Smoking is relevant in trauma surgery, and a way to prevent presurgical smoking has not been noted; hence, the risk of nonunion is high in these patients. However, exactly how it hinders fracture healing is unknown, but it has been proposed that it causes vasoconstriction and hypoxia. The main substance responsible for this is nicotine, which stimulates the release of catecholamines that cause vasoconstriction [4]. Thus, this study aims to assess the influence of smoking in the healing of tibial fractures following fixation.

## Materials and methods

This study is a retrospective study of 110 cases of admission and operation in the Department of Orthopedics at RL Jalappa Hospital, Tamaka, Kolar, from July 2017 to January 2021. Of the patients, 55 were smokers and 55 were nonsmokers. Approval for the study was obtained from the institutional ethics committee, and informed consent was obtained from all patients when called for follow-up. All surgeries were conducted at the institute of the current study. The inclusion criteria for our study were patients with closed tibial shaft fractures, age group between 18 and 60 years old, and smokers and nonsmokers operated for tibial shaft fractures. The exclusion criteria were patients with metabolic bone disease, treatment delay of >3 weeks, patients with HIV infection, patients with pathological fractures, polytrauma patients, and patients with tibial fractures with bone loss.

Method of fracture stabilization

The method of fracture fixation was based on the surgeon’s preference. Two methods of fixation were used: intramedullary interlocking (IMIL) nailing and MIPO. The fixation method was not related to the patients’ smoking status.

Clinical parameters

Patients who smoked before the trauma and continued to smoke after fracture fixation following discharge and those who had a smoking index of >400 were classified as smokers [5]. Fracture union was assessed following the time to full weight-bearing mobilization without pain at the fracture site.

Radiographic parameters

Fracture location was classified into three types: proximal, middle, and distal third tibial shaft fractures. Immediate postoperative radiographs were taken to analyze fracture reduction. Follow-up radiographs were taken (anteroposterior (AP) and lateral views), and fracture healing was analyzed following the number of healed cortices (four cortices). Adequate callus formation was determined using the callus index, which was calculated as the maximum diameter of the callus divided by the diameter of bone. Union was said to be achieved if at least three of four cortices were healed. Radiographic time to union was analyzed in weeks.

Statistical analysis

Data were entered using Microsoft Excel (Microsoft®, Redmond, WA, USA) and analyzed using the Statistical Package for the Social Sciences version 20 (IBM, Armonk, NY, USA). All continuous variables were summarized using mean (standard deviation (SD)) or median (interquartile range (IQR)) depending on the normality of the distribution. Categorical variables were summarized using proportions. Comparison of categorical outcomes (age group, gender, fixation type, site, mode of injury, comorbidity, infection, and outcomes (e.g., nonunion, malunion, and delayed union)) across study groups (smokers versus nonsmokers) was done using the chi-square test.

## Results

All patients were available for follow-up and were followed up at months 1, 3, and, 6 and year 1. Table 1 describes the study participants by their smoking status. This study only included males because the rural female population showed no smoking history.

**Table 1:** Description of the study participants by smoking status (n = 110) n: total number of patients, SD: standard deviation

Parameter	Smoker (n = 55)	Nonsmoker (n = 55)	p-value
Mean (SD) age, in years	46.8 (10.4)	43 (9.4)	0.051
Age category (in years)			
22–30	3 (5.5%)	5 (9.1%)	0.528
31–40	15 (27.3%)	17 (30.9%)	
41–50	15 (27.3%)	18 (32.7%)	
51–60	22 (40%)	15 (27.3%)	
Gender			
Male	55 (100%)	55 (100%)	-

**Figure 1:**
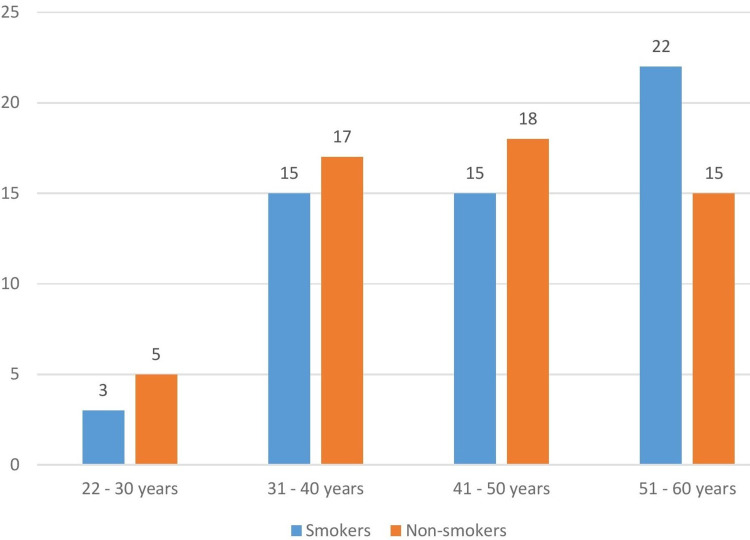
Age distribution (n = 110)

The mean age of the patients who smoked and did not smoke was 46.8 and 43 years old, respectively. The age distribution of the study population is shown in Figure 1, where the majority of the patients who are smokers and nonsmokers were 51-60 (40%) and 41-50 (32.7%) years old, respectively.

Table 2 shows that the right leg was predominantly affected in both smokers (34 patients, 61.8%) and nonsmokers (32 patients, 58.2%). With treatment, the majority of smokers (41 patients, 74.6%) and nonsmokers (42 patients, 76.4%) underwent IMIL nailing.

**Table 2:** Description of disease compared between smokers and nonsmokers (n = 110) n: total number of patients, IMIL: intramedullary interlocking, MIPO: minimally invasive percutaneous plate osteosynthesis

Parameter	Smoker (n = 55)	Nonsmoker (n = 55)	p-value
Affected side			
Left	21 (38.2%)	23 (41.8)	0.697
Right	34 (61.8%)	32 (58.2%)	
Fixation type			
IMIL nail	41 (74.6%)	42 (76.4%)	0.825
MIPO	14 (25.4%)	13 (23.6%)	
Fracture location			
Distal	16 (29.1%)	14 (25.4%)	0.567
Middle	38 (69.1%)	38 (69.1%)	
Proximal	1 (1.8%)	3 (5.5%)	

**Figure 2:**
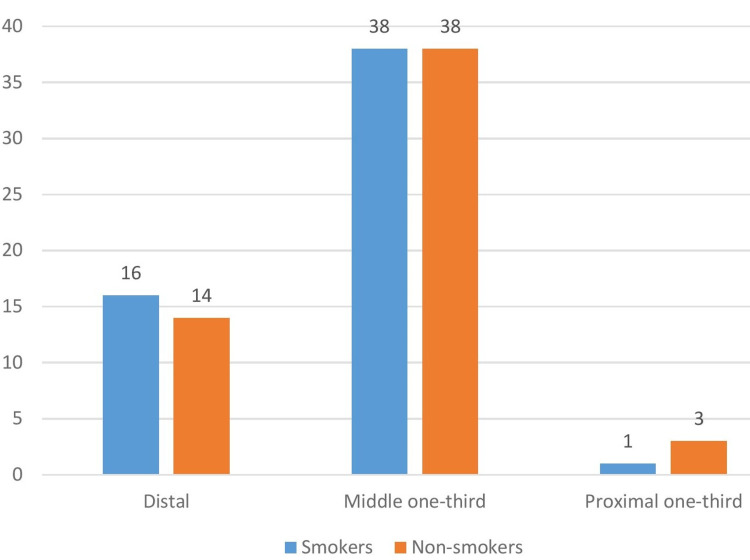
Fracture distribution (n = 110)

The majority of the fractures were noted in the middle third region in both groups (Figure 2).

Clinical and radiographic follow-up one month after fracture treatment showed adequate callus formation in 26 (47.3%) and 52 (94.5%) patients who were smokers and nonsmokers, respectively. No callus formation was observed in nine smokers at the end of one month. This discrepancy was determined to be attributable to a greater proportion of patients who smoked after hospital.

Table 3 shows the follow-up observations in both groups. Within the group of patients who are smokers, five (9.1%), 15 (27.3%), and 23 (41.8%) patients showed healing of three cortices, two cortices, and only one cortex, respectively, at the end of three months. Twelve (21.8%) patients in this group showed no callus formation even after three months. When compared with nonsmokers, the majority (96.4%) of patients showed healing of three cortices, and 3.6% showed healing of two cortices (p < 0.001).

**Table 3:** Comparison of fracture healing between smokers and nonsmokers (n = 110) a: six of 16 and 14 of 18 patients in the smoking group had signs of infection n: total number of patients

	Parameter	Smoker (n = 55)	Nonsmoker (n = 55)	p-value
1 month	Adequate callus formation	26 (47.3%)	52 (94.5%)	<0.001
	Minimal callus formation	20 (36.4%)	3 (5.5%)	
	No callus formation	9 (16.4%)	0	
3 months	Three healed cortices	5 (9.1%)	53 (96.4%)	<0.001
	Two healed cortices	15 (27.3%)	2 (3.6%)	
	One healed cortex	23 (41.8%)	0	
	Callus formation	12 (21.8%)	0	
6 months	Healed	0	53 (96.4%)	<0.001
	Three healed cortices	15 (27.3%)	2 (3.6%)	
	One or two healed cortices	28 (40.9%)	0	
	Callus formation	7 (12.7%)	0	
	No progression	5 (9.1%)	0	
1 year	Healed	8 (14.6%)	55 (100%)	<0.001
	Three healed cortices	16^a^ (29.1%)	0	
	One or two healed cortices	18^a^ (32.8%)	0	
	Nonunion	13 (23.6%)	0	

**Figure 3:**
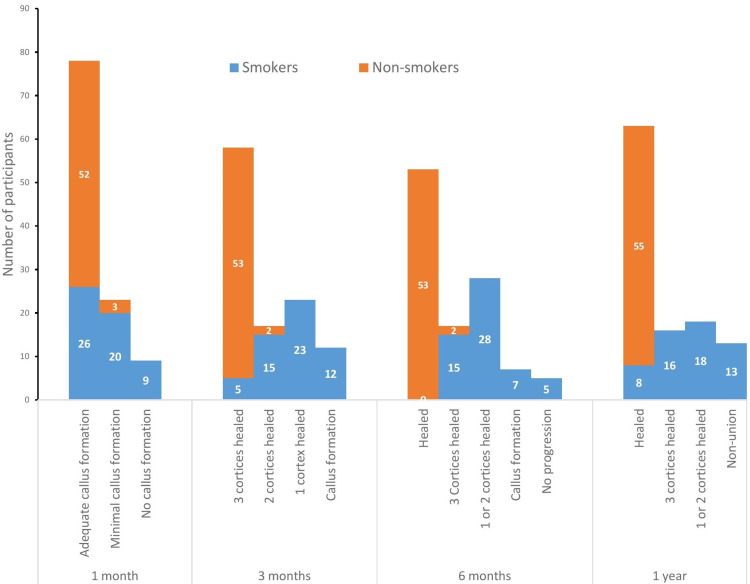
Fracture healing between smokers and nonsmokers (n = 110)

After one year of follow-up, 13 smokers had nonunion (23.6%) and all the 55 patients who were not smokers had fracture union (Figure 3). These patients with nonunion were further treated with either exchange nailing/plating and bone grafting or bone marrow injection at the nonunion site.

**Figure 4:**
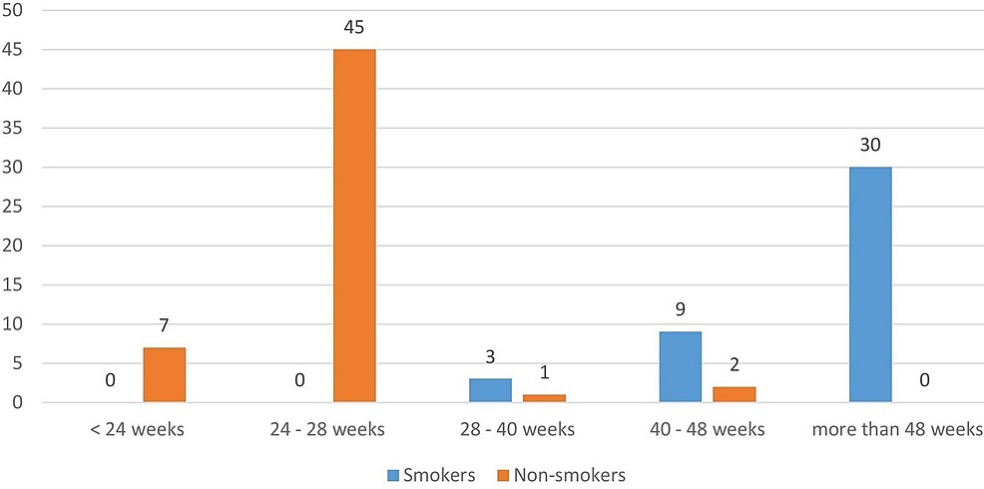
Time to fracture healing between smokers and nonsmokers (n = 110)

Table 4 shows the time to fracture healing in both groups. The mean time of healing in smokers and the average time to union in nonsmokers were >48 and 24 weeks, respectively. The majority (54.6%) of smokers took >48 weeks to heal, whereas 81.8% of patients in the nonsmoking group took 24-28 weeks to heal (p < 0.001). This shows that time to fracture union was significantly longer in smokers when compared with nonsmokers (Figure 4).

**Table 4:** Comparison of time to fracture healing between smokers and nonsmokers (n = 110) n: total number of patients, IQR: interquartile range

Parameter	Smoker (n = 55)	Nonsmoker (n = 55)	p-value
Occurrence of fracture union	42 (76.4%)	55 (100%)	<0.001
Nonunion	13 (23.6%)	0	
Time for fracture healing			
Median time for healing	>48 weeks	24 weeks	
IQR	48–48 weeks	24–25 weeks	
Frequency of healing duration			
<24 weeks	0	7 (12.7%)	<0.001
24–28	0	45 (81.8%)	
28–40	3 (5.4%)	1 (1.8%)	
40–48	9 (16.4%)	2 (3.6%)	
>8 weeks	30 (54.6%)	0	
Nonunion	13 (23.6%)	0	

## Discussion

Researchers have long suspected that smoking interferes with healing. The theory states that it adversely affects bone mineral density and the dynamics of bone and wound healing [6]. Some studies suggest that TGF-beta 1 increases after surgery and serves as a marker for healing. Smokers have been observed to have low TGF-beta 1 levels than nonsmokers during the first four weeks after surgery, providing an explanation for delayed union in these patients [7]. In a study conducted in 2012, Al-Hadithy et al. observed that the precise role of nicotine in fracture healing is uncertain and recommended that patients should attempt smoking cessation therapy before any elective orthopedic procedure [8].

Patel et al. conducted a meta-analysis in 2013 and observed that smoking significantly affected fracture union, especially in tibial shaft fractures and also in spinal, foot, and ankle fusions. They observed that smokers have a 40% increased time to union and chance of nonunion when compared with nonsmokers [9].

In addition, Harvey et al. conducted a retrospective study and found that smokers and nonsmokers had a union rate of 84% and 94%, respectively, in open tibial shaft fractures. The authors observed that the average number of weeks until union in patients who smoked and did not smoke was 52.3 to 44.1 weeks, respectively, with open tibial shaft fractures, which were not statistically significant [10]. This study shows that open tibial fracture healing can be influenced by several factors (e.g., wound size, infection, and soft tissue injury) and delayed healing cannot be solely attributed to smoking. In the current study, 65.8% of smokers with tibial shaft fractures fixed with IMIL nailing had delayed union, which is significantly higher when compared to a study conducted by Manon et al., where delayed union occurred in 22.8% of patients who were smokers with tibial shaft fractures fixed with IMIL nailing [11].

Anoop et al. conducted a retrospective study of 150 patients, in which 75 tibias showed nonunion (72.4% smokers). The author observed that smoking has a strong effect on bone healing. Proper smoking history should be taken, and its implications should be explained to the patient [12]. In addition, 13 tibias showed nonunion in all patients who were smokers when compared to the current study of 110 patients.

**Figure 5:**
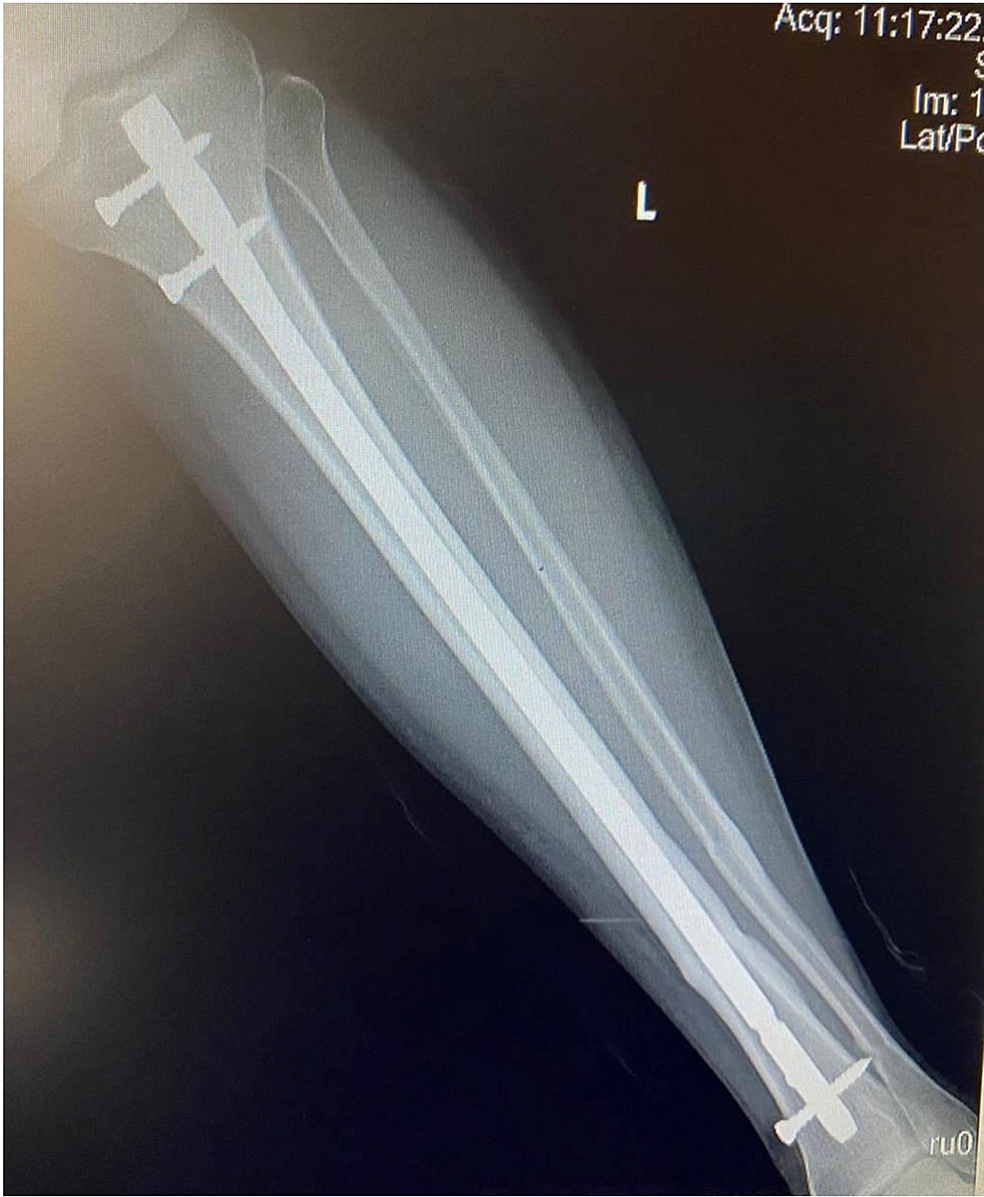
Six months follow-up AP radiograph of a 34-year-old nonsmoker showing united distal third fracture of the left tibia with IMIL nail in situ

**Figure 6:**
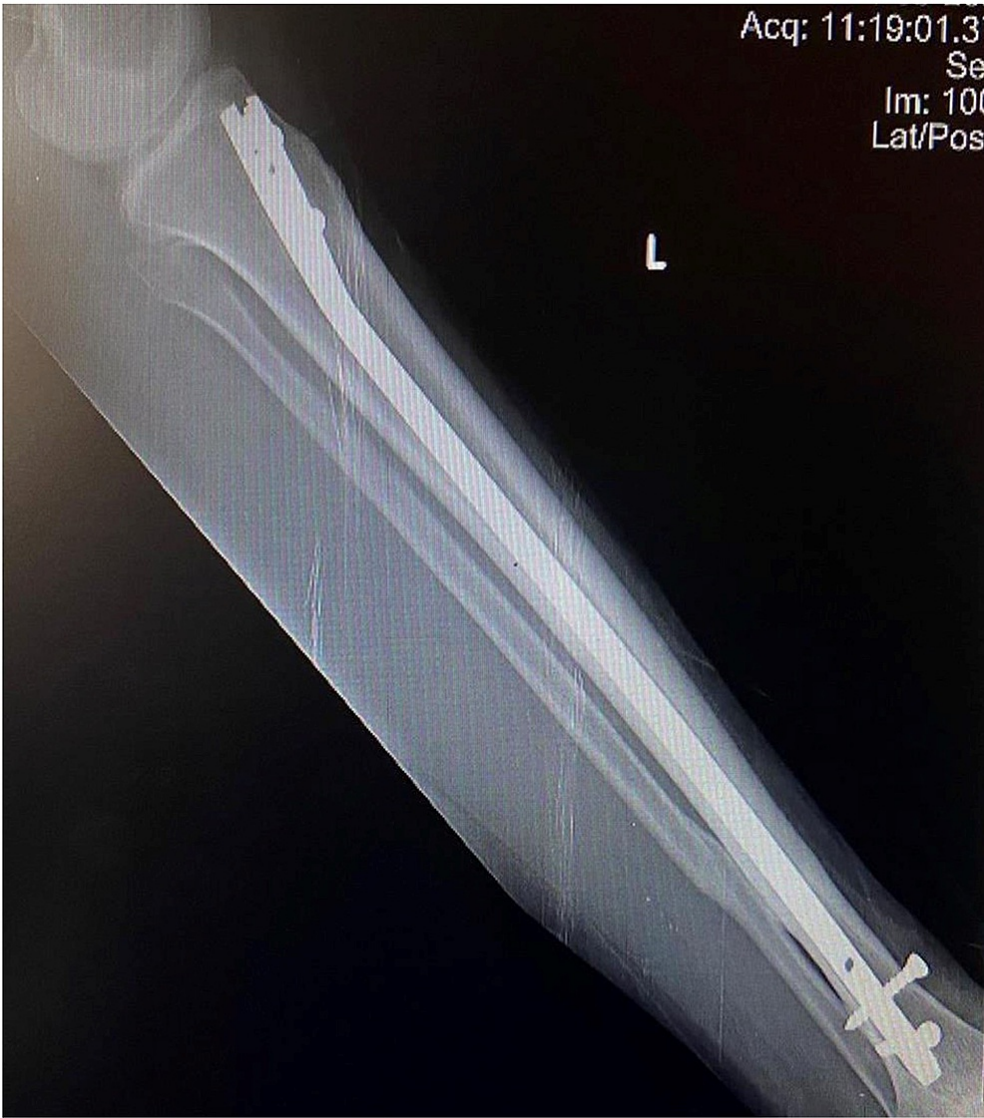
Six months follow-up lateral radiographs of a 34-year-old nonsmoker showing united distal third fracture of the left tibia with IMIL nail in situ

Schmitz et al. conducted a study of closed and grade I open tibia shaft fractures treated with a cast, external fixation, or IMIL nailing over four years. They observed that the median time to clinical healing in smokers was 289 days when compared with 136 days in nonsmokers [13]. In one systematic review, the risk of long bone fracture nonunion was found to be 12% higher in smokers than in nonsmokers, and the mean fracture healing time was 30.2 weeks in smokers versus 24.1 weeks in nonsmokers [14]. The current study has measured time to radiological union in weeks where it was observed that smokers took >48 weeks to heal when compared with nonsmokers who took 24 weeks to heal (Figures 5, 6).

**Figure 7:**
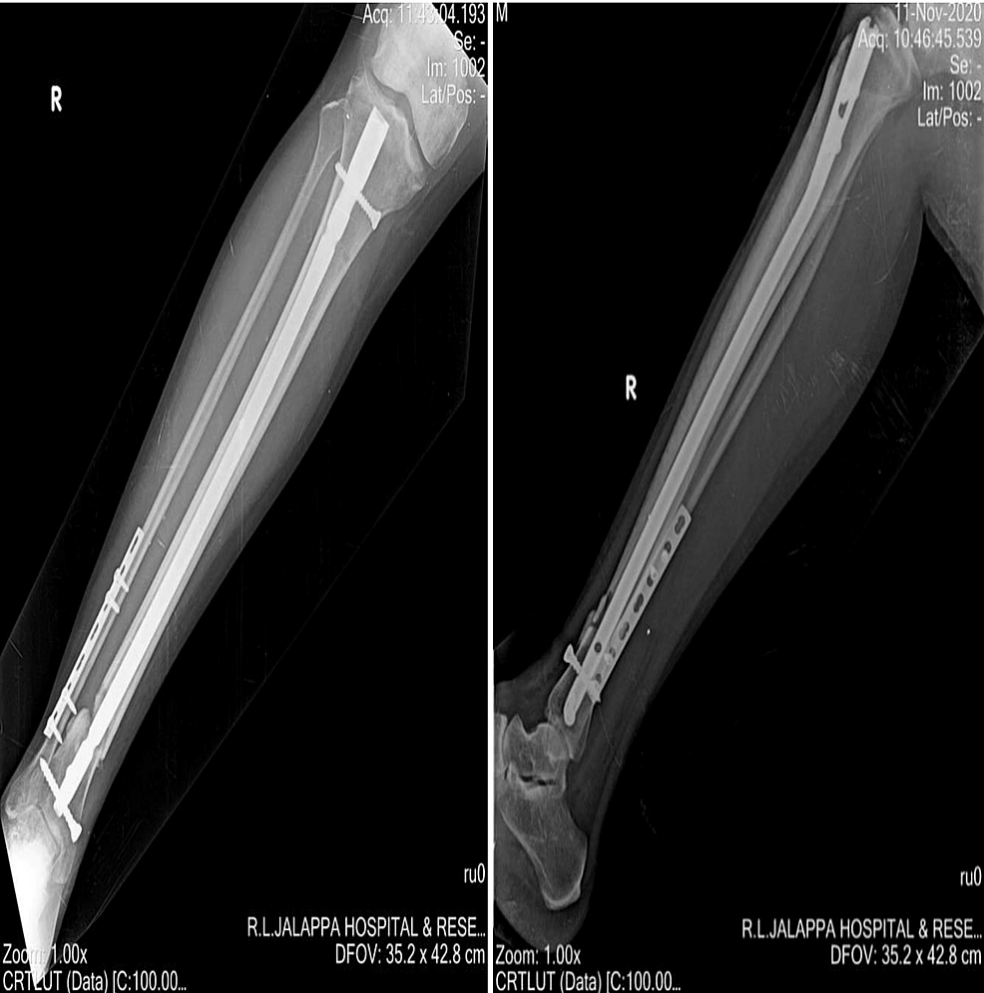
Nine months follow-up AP and lateral radiographs of a 36-year-old smoker showing nonunion of the distal third fracture of the right tibia with IMIL nail in situ

According to Bender et al., the risk of delayed union was higher in current and previous smokers when compared with nonsmokers after studying the harmful effects of smoking in a study of 85 patients with closed and open tibial shaft fractures [4]. Adams et al. noted that the average time to union in patients with open tibial fractures was 32 (smokers) and 28 (nonsmokers) weeks [15]. Secondary procedures (e.g., bone grafting and exchange nailing) to stimulate union were required in smokers when compared with nonsmokers. The current study has excluded open fractures as delayed union, and infection development is multifactorial in these patients. Castillo et al. reported that previous smokers were at increased risk of delayed union after tibia fractures, but their risk was not as great as current smokers. This study also showed that current and previous smokers were 37% and 32% less likely to achieve union than nonsmokers, respectively (Figure 7). Current smokers were more than twice as likely to develop an infection and 3.7 times as likely to develop osteomyelitis. Previous smokers were 2.8 times as likely to develop osteomyelitis but were at no greater risk for other infection types [16]. In the current study, 20 (36.4%) smokers were observed to developed infection, whereas none of the nonsmokers showed any signs of infection at the end of one year.

In a study of 118 patients with open tibial fractures, smokers had a significantly higher incidence of osteitis (27% compared to 9%) and a significantly longer time until union (i.e., a mean time of 33 compared to 26 weeks in nonsmokers) [17].

Current and previous smokers required more time to heal, were absent longer from work, and had a significantly poorer functional outcome according to the Merchant and Dietz score and the Lysholm score [18]. Although smoking cessation has been routinely advised perioperatively, current guidelines are vague because conclusive studies regarding this are scarce. The few published recommendations are variable and state that smoking cessation should commence at 1-21 and 5-28 days preoperatively and postoperatively, respectively [19]. Five smoke-free days post-surgery has been documented as having a favorable outcome on wound healing [20]. One week was implicated by Lind et al. due to the pharmacokinetics of free radicals and thrombotic components [21]. Whitesides et al. showed that nonsmokers took two months to produce 1 cm of bone, whereas smokers took three months to grow a similar amount of tissue [19]. Lau et al. suggested that the effect of smoking on union is exerted by bone resorption at the fracture site [22].

## Conclusions

In conclusion, smoking hinders fracture healing post-surgical fixation, and smokers have a higher chance of developing surgical site infection and osteomyelitis. Smokers take a longer time to achieve radiological union and also have a high chance of delayed union and nonunion when compared with nonsmokers. Postoperative smoking cessation is as important as preoperative smoking cessation, and patients should be strictly counseled regarding the same.

The current study has certain limitations. The current study included only male patients. Many factors including age, high- or low-energy trauma, and associated soft tissue injuries with trauma affect fracture healing; hence, further randomization would have been more appropriate. Only closed tibial shaft fractures have been considered, as an increased risk of delayed union and nonunion in patients with open tibial fractures were noted in smokers and because other risk factors for the aforementioned exist (e.g., the open wound itself). In addition, the sample size is inadequate to reach a definitive conclusion.
